# Smart Data Collection for the Assessment of Treatment Effects in Irritable Bowel Syndrome: Observational Study

**DOI:** 10.2196/19696

**Published:** 2020-11-02

**Authors:** Zsa Zsa R M Weerts, Koert G E Heinen, Ad A M Masclee, Amber B A Quanjel, Bjorn Winkens, Lisa Vork, Paula E L M Rinkens, Daisy M A E Jonkers, Daniel Keszthelyi

**Affiliations:** 1 Division Gastroenterology-Hepatology, Department of Internal Medicine NUTRIM School for Nutrition and Translational Research in Metabolism Maastricht University Medical Center+ Maastricht Netherlands; 2 MEMIC Center for Data and Information Management Maastricht University Maastricht Netherlands; 3 Department of Methodology and Statistics Maastricht University Medical Center+ Maastricht Netherlands; 4 Care and Public Health Research Institute Maastricht University Medical Center+ Maastricht Netherlands

**Keywords:** irritable bowel syndrome, digital diary, smartphone application, mobile phone application, mhealth, e-health, compliance, electronic case report file, patient reported outcome measures, peppermint oil, PERSUADE study.

## Abstract

**Background:**

End-of-day symptom diaries are recommended by drug regulatory authorities to assess treatment response in patients with irritable bowel syndrome. We developed a smartphone app to measure treatment response.

**Objective:**

Because the employment of an app to measure treatment response in irritable bowel syndrome is relatively new, we aimed to explore patients’ adherence to diary use and characteristics associated with adherence.

**Methods:**

A smartphone app was developed to serve as a symptom diary. Patients with irritable bowel syndrome (based on Rome IV criteria) were instructed to fill out end-of-day diary questionnaires during an 8-week treatment. Additional online questionnaires assessed demographics, irritable bowel syndrome symptom severity, and psychosocial comorbidities. Adherence rate to the diary was defined as the percentage of days completed out of total days. Adherence to the additional web-based questionnaires was also assessed.

**Results:**

Overall, 189 patients were included (age: mean 34.0 years, SD 13.3 years; female: 147/189, 77.8%; male: 42/189, 22.2%). The mean adherence rate was 87.9% (SD 9.4%). However, adherence to the diary decreased over time (*P*<.001). No significant association was found between adherence and gender (*P*=.84), age (*P*=.22), or education level (lower education level: *P*=.58, middle education level: *P*=.46, versus high education level), while higher anxiety scores were associated with lower adherence (*P*=.03). Adherence to the online questionnaires was also high (>99%). Missing data due to technical issues were limited.

**Conclusions:**

The use of a smartphone app as a symptom diary to assess treatment response resulted in high patient adherence. The data-collection framework described led to standardized data collection with excellent completeness and can be used for future randomized controlled trials. Due to the slight decrease in adherence to diary use throughout the study, this method might be less suitable for longer trials.

## Introduction

Irritable bowel syndrome (IBS) is a highly prevalent chronic disorder of brain-gut interaction characterized by recurrent abdominal pain and altered bowel habits [1]. Since well-defined organic causes and validated biomarkers for IBS are lacking, patient-reported outcome measures are crucial in assessing treatment response. Accordingly, drug regulatory authorities currently recommend using end-of-day symptom scores in IBS trials to measure drug efficacy [2,3]. Diaries are generally considered to be suitable to measure end-of-day gastrointestinal symptom scores and have the ability to capture symptom variability over time [4]. The validity and reliability of paper diaries, however, may be impeded by fake adherence [5] (ie, falsifying or backfilling written answers outside of the proposed time window [6]). The gap between reported and actual adherence to paper diaries has been shown to be as large as 80% in some studies [7]. Because backfilling introduces considerable recall and ecological bias [8], using paper diaries can distort trial results, which can ultimately lead to incorrect conclusions about treatments. Efficacy endpoints in clinical trials should, therefore, preferably not be assessed by paper diaries.

Recent technological advancements and the widespread availability of smartphones have given rise to numerous health-related apps and electronic diaries in the last decade [9-12], both in clinical and research settings. Digitalized data collection provides several advantages over a paper-based data collection as it results in higher data entry quality and more efficient data handling [13]. For example, responses can be verified automatically by built-in response requirements, routing, and data validation, and manual data transcription can be omitted. More importantly, data entry for previous days can be prevented, and all entries can be given a date- and time-stamp, generating more valid (momentary) results and allowing assessment of actual adherence to the diary. Studies that have implemented electronic diaries have reported excellent adherence, ranging from 76%-100% [5,14,15].

These advantages encouraged our group to implement a digital data-collection framework and develop a smartphone app that can be used as a digital symptom diary. This diary was used to collect Food and Drug Administration (FDA) recommended efficacy outcomes in our randomized placebo-controlled clinical trial on the efficacy of peppermint oil in IBS called the PERSUADE study [16]. This observational study describes the development and evaluates the performance of the overall digital framework used for data collection in that clinical trial. Within the realm of IBS trials, the use of a digital symptom diaries is relatively new; most previous studies have not reported adherence for the assessment method used, and data on adherence in other populations cannot necessarily be extrapolated to IBS. Therefore, our primary aim was to evaluate the performance of a custom-made digital symptom diary in patients with IBS, in particular by assessing patients’ adherence. Since patient characteristics can impact adherence [17,18], our secondary aim was to identify sociodemographic and clinical patient characteristics associated with adherence.

## Methods

### Overview

The study was based on data from the PERSUADE study [16]. This was a randomized double-blind placebo-controlled trial (clinicaltrials.gov; NCT02716285) conducted in 4 hospitals located throughout the Netherlands (Multimedia Appendix 1; Figure S1). The study protocol was approved by the Maastricht University Medical Center+ Ethics Committee. All study procedures were performed in compliance with Good Clinical Practice Guidelines and according to the revised Declaration of Helsinki [19]. All participants gave written informed consent prior to participation.

The study design of the PERSUADE has been described in detail elsewhere [16]. In brief, the primary aim was to investigate the efficacy of peppermint oil—a conventional small-intestinal release formulation and a novel ileocolonic release formulation—in patients with IBS. To this end, patients between 18-75 years of age, who fulfilled the Rome IV criteria for IBS and had a mean worst abdominal pain score of at least 3 on an 11-point rating scale (0, no pain; 10, worst possible pain) during a 14-day pretreatment period were included. Participants were randomized to placebo, small-intestinal release peppermint oil, or ileocolonic release peppermint oil for an 8-week treatment period.

Data were collected using a customized framework for digital data collection, specifically designed and developed for the trial, consisting of (1) a digital symptom diary (smartphone app); (2) an electronic case report file (eCRF, Castor EDC); (3) web-based patient questionnaires (Castor EDC); and (4) a planning tool (Ldot). During the 14-day pretreatment and the 8-week treatment period, patients were instructed to register symptoms daily in the digital symptom diary. Study visits and telephone follow-up telephone interviews were documented in the eCRF. Patients were asked to complete several web-based questionnaires at different time-points within the study duration. The complete list of inclusion criteria and study overview with timing of the questionnaires is given in the Multimedia Appendix 1. Primary efficacy results of the PERSUADE study have been described elsewhere [16].

### Digital Symptom Diary: Smartphone App

For the digital symptom diary, an electronic smartphone app was developed by the Center for Data and Information Management at Maastricht University (MEMIC), in close collaboration with the investigators. The app was programmed using Xamarin, a framework to develop cross-platform apps using C sharp programming. The PERSUADE app supports Android and iOS devices. A Maastricht University industrial designer designed the visual content. A MEMIC team of data managers and researchers of the Maastricht University Medical Center+ Neurogastroenterology group tested the app and provided feedback throughout several phases of development. Additionally, a patient was asked to use the diary and provide feedback regarding its user friendliness. Patient inclusion commenced once a version was reached that all agreed on.

**Figure 1:**
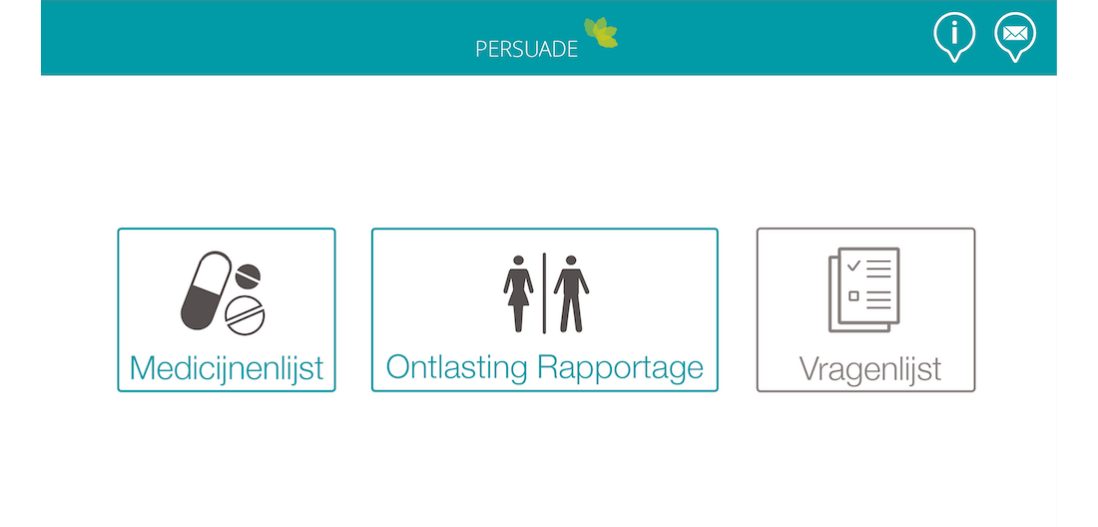
Home screen of the smartphone app.

**Figure 2:**
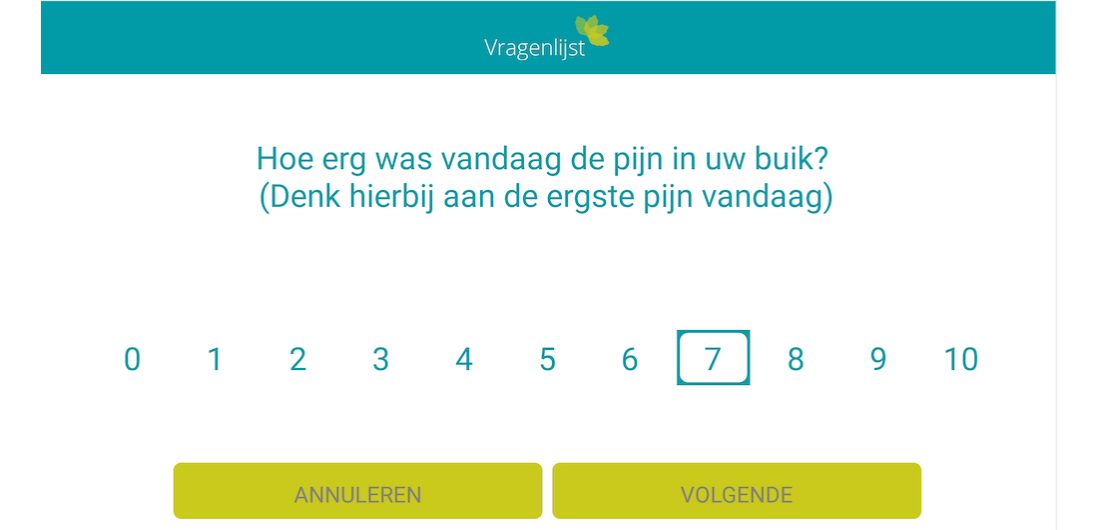
Primary outcome measure.

The app’s home screen consisted of 3 main elements: the daily end-of-day symptom questionnaire, a medication list, and the Bristol stool chart questionnaire (Figure 1). The end-of-day symptom questionnaire included one main question to assess the primary outcome (in accordance with FDA guidelines): “How would you rate your abdominal pain today? Think about the worst abdominal pain today” (11-point numerical rating scale) (Figure 2). The daily symptom questionnaire was accessible between 6 PM and 12 PM and was unavailable outside this time window, to avoid premature completion. Other daily questions were related to “need of rescue medication” and “adverse events experienced.” If a patient had not completed the daily entry before 10 PM of that particular day, one push notification was sent. At the end of each week, the end-of-day questionnaire consisted of additional questions regarding abdominal discomfort, abdominal bloating, abdominal cramping, belching, nausea, and urgency during the last week (11-point scale numerical rating scale). It was not possible to enter data for previous days, and participants could not review prior entries. Automated routing, response requirements, and real-time data verification were built in to increase data quality and completeness.

The medication list was used once to register all regular medications. Patients were asked to keep their concomitant medication use as stable as possible. However, if alterations were needed, they were able to delete, add, or change the dosage of nongastrointestinal drugs.

**Figure 3:**
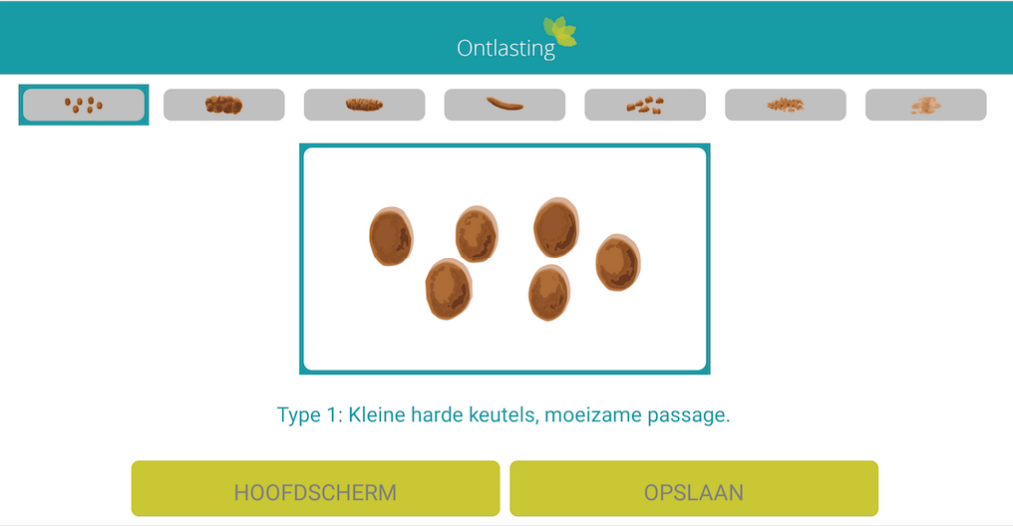
Bristol Stool Form Scale.

The Bristol stool form scale was used to register all bowel movements (Figure 3). There was no minimum or maximum number of registrations per day.

All patients received extensive verbal and written instructions during the screening visits on how to use the app and were encouraged to contact the researchers if the app crashed or otherwise did not function properly. A personalized username and password were provided for access to the app. Patients were instructed to enable automatic updates of the app to ensure the most recent version was used. If a patient did not own a smartphone or tablet, a device was provided. During the complete pretreatment and treatment periods, an alert system in the planning tool notified the investigators when a patient had failed to submit 3 or more daily entries. In addition, the development team received automated notifications of app crashes.

### Web-Based Patient Questionnaires

At randomization, at 2, 4, 6, and 8 weeks of treatment, and at 3 and 6 months of follow-up after treatment ended, patients were asked to fill out web-based questionnaires. We chose not to implement these into the digital diary because of the large number of questions. Included were a questionnaire regarding demographics and lifestyle and validated questionnaires regarding symptom severity (IBS Symptom Severity System), quality of life (IBS Quality of Life, EuroQoL EQ-5D-5L), comorbid symptoms of anxiety and depression (General Anxiety Disorder 7, Patient Health Questionnaire 9), and health care utilization and productivity loss (Medical Consumption Questionnaire, Productivity Cost Questionnaire). Patients received invitations via email containing an HTML-link to the electronic environment. If a patient had not completed the questionnaire within 2 days, 2 automatic reminders were sent via email. Automated routing, response requirements, and real-time data verification were built in to increase data-quality and completeness.

### eCRF

During the study visits and telephone follow-up calls, investigators documented all findings in a cloud-based eCRF. The eCRF forms were built by the first author with input from the other authors and contained items regarding demographics, Rome IV diagnostic criteria for IBS, history and physical examination, adverse events, and general wellbeing. Investigators were given unique usernames and passwords to view and add data for their respective inclusion centers. To achieve registration uniformity, the investigators were trained on how to enter data, and additional step-by-step instructions were given in a standard operating procedure document. Real-time automated data verification and corresponding pop-up notifications were built in to prevent typing errors or other erroneous entries. Automated routing of questions and response requirements ensured that correct items were displayed and filled in. An audit trail enabled tracking of all data changes.

### Ldot Planning Tool

Ldot is a web-based tool developed by the Center for Data and Information Management at Maastricht University and was used to monitor study logistics. All personal patient data were entered into Ldot, and the app supported the study workflow by indicating when each study event (eg, randomization, follow-up call, etc) needed to take place for each patient. Ldot was able to communicate with the digital diary and the web-based questionnaires. For example, all email invitations for the questionnaires were sent automatically via Ldot. Patients’ adherence to the diary and web-based questionnaires could be monitored within Ldot and investigators were notified if patients failed to complete 3 consecutive days in the diary. Investigators were also notified if patients failed to complete a web-based questionnaire after a reminder was given. To guarantee the anonymity and quality of research data, no research data could be entered into Ldot. Investigators could view and add personal data for their respective inclusion centers. There was no possibility of viewing data from other inclusion centers, except for the coordinating investigator (first author) who had access to all data. An audit layer of the app tracked and stored information of all changes.

### Storage, Servers, and Privacy

All software and data storage complied with international ISO27001, ISO9001, good clinical practice guidelines, and Dutch NEN7510 guidelines. Electronic diary data, web-based-questionnaire data, eCRF data, and sensitive personal data (Ldot) were all stored on different (nonconnected) servers. Several back-ups were made per day. Access to the servers was and will be restricted, with 24-hour on-site surveillance. Data will be stored for 15 years after study completion.

### Outcome Measures

The primary outcome of the current study was patients’ adherence to the digital symptom diary, defined as the mean percentage of entries and calculated by dividing the number of completed entries by the number of minimal requested entries (total number of days in study). Patients were instructed to complete a diary entry on all consecutive days during the 14-day pretreatment and 56-day treatment period, or all days until discontinuation with the study.

Secondary outcomes were change in mean adherence per week over time, sociodemographic characteristics, clinical patient characteristics associated with adherence, time of diary completion, and difference in adherence between patients who were defined as responders to treatment versus nonresponders. Potential data loss and critical evaluation points were considered to explore the overall feasibility of a smartphone app as a primary data-collection tool in a randomized controlled trial. Other secondary outcomes were patients’ adherence to and completeness of the additional web-based questionnaires and investigators’ adherence to and completeness of the eCRF.

### Statistical Analysis

Statistical analyses were carried out using SPSS statistical software (version 25.0 for Macintosh; IBM Corp). Data are expressed as mean and standard deviation or as number and percentage. Multivariable linear regression analysis was used to investigate the association between baseline patient characteristics and adherence to the digital diary, adjusting for minimization variables (age, gender, IBS subtype, inclusion center, and treatment group). A repeated measures analysis of variance was performed to assess the influence of time (weeks) on adherence. If the Mauchly test indicated that the sphericity assumption was not met, Greenhouse-Geisser corrected results were reported. A *P*<.05 (2-sided) was considered statistically significant.

## Results

### General

Overall, 190 patients were randomized. One patient was randomized erroneously (ie, without fulfilling all inclusion criteria). Therefore, 189 patients (age: mean 34.0, SD 13.3 years; female: 147/189, 77.8%; male: 42/189, 22.2%) were analyzed (n=64 in the placebo group, n=62 in the small intestinal release peppermint group, n=63 in the ileocolonic release peppermint oil group). Of the 189 patients, 95.8% (181) were Caucasian and 4.2% (8) were of mixed descent. Most patients (109/189, 57.7%) were recruited from a primary care setting. Eleven patients withdrew from the study during the treatment period (data until discontinuation were included in the analyses). Baseline characteristics are presented in Table 1. During recruitment, only a single patient stated the digital data collection as a reason not to participate.

**Table 1:** Summary of patient demographic and baseline characteristics.

Characteristic (N=189)		Value
**Age (years)**		
	Mean (SD)	34.0 (13.3)
	Range	18-70
**Gender, n (%)**		
	Male	42 (22.2)
	Female	147 (77.8)
**Education level, n (%)**		
	No education	1 (0.5)
	Low	15 (7.9)
	Moderate	80 (42.3)
	High	93 (49.2)
**Setting, n (%)**		
	Primary care	109 (57.7)
	Secondary care	41 (21.7)
	Combined secondary & tertiary care	39 (20.6)
**IBS^a^-subtype^b^, n (%)**		
	Diarrhea	83 (43.9)
	Constipation	42 (22.2)
	Mixed	40 (21.2)
	Undefined	24 (12.7)
**IBS severity^c^**		
	Score, mean (SD)	276.5 (71.9)
	Mild, n (%)	15 (7.9)
	Moderate, n (%)	100 (52.9)
	Severe, n (%)	74 (39.2)
IBS Quality of Life score^d^, mean (SD)		73.0 (15.1)
EQ-5D-5L utility score^e^, mean (SD)		0.7 (0.2)
**Psychological comorbidities^f^, mean (SD)**		
	Anxiety	5.4 (4.3)
	Depression	6.8 (4.5)

^a^IBS: irritable bowel syndrome.

^b^Determined in a face-to-face interview (according to Rome IV criteria).

^c^The Irritable Bowel Syndrome (IBS) Symptom Severity System consists of 5 items with a maximum score of 100, higher scores indicate more severe symptoms.

^d^The IBS Quality of Life questionnaire consists of 34 items with a 5-point Likert scale (1=good, 5=worse).

^e^The EuroQol-5D-5L measures 5 dimensions of quality of life. Raw scores are transformed to utility scores [20], which vary from 1 (perfect health) to 0 (death).

^f^The General Anxiety Disorder–7 consists of 7 items with a 4-point response scale (0=not at all, 3=almost every day). The Patient Health Questionnaire–9 consists of 9 items with a 4-point response scale (0=not at all, 3=almost every day).

### Patients’ Adherence to the Digital Symptom Diary

**Figure 4:**
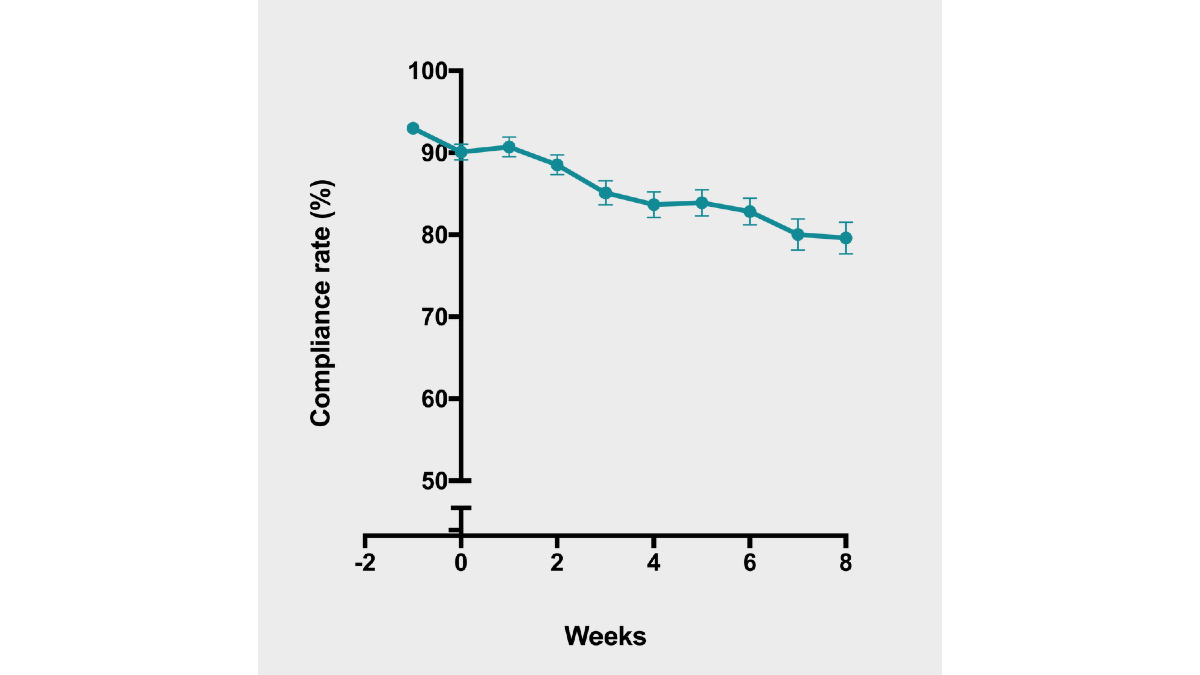
Adherence.

Most patients used their own smartphones, but 4 out of 189 patients needed a device provided by the investigators. Patient adherence to the daily digital symptom diary was excellent during the entire study period, reflected by a mean completion rate of 87.9% (SD 9.4%), 91.5% (SD 9.2%), and 86.9% (SD 10.8%), during all 70 days of study duration, the 14-day pretreatment period, and the 8-week treatment period, respectively. Adherence during the treatment period did not differ significantly for treatment groups compared to that of the placebo (placebo: mean 87.2%; small-intestinal release: 88.3%; *P*=.67), and for the ileocolonic release peppermint oil (mean 87.2%; *P*=.33). Adherence did not differ between patients that were clinical responders to treatment (mean 88.0%) versus patients who were nonresponders (mean 86.2%). Over the complete study period of 70 days, a significant decrease in mean weekly patient adherence to the end-of-day questionnaire was found (*F*_5.9, 1114.9_=15.5, *P*<.001) (Figure 4). Nevertheless, adherence was still good at the end of the study (mean 79.6%, SD 26.6%) (Figure 4).

When exploring independent baseline predictors for adherence, the combined regression model that included all minimization variables (age, gender, IBS subtype, inclusion center), treatment group, baseline IBS symptom severity, anxiety and depression scores, and education level showed that 1 of the 4 inclusion centers (center C; Multimedia Appendix 1, Figure S2), (regression coefficient B –10.04, 95% CI –19.51 to –0.56, *P*=.04) and anxiety scores at baseline (B –0.59, 95% CI –1.12 to –0.06, *P*=.03) were negatively associated with adherence throughout the study. Indeed, when comparing adherence between the different inclusion centers, adherence in center C was lowest (mean 82.3%, SD 12.5%), compared with adherence in centers A (mean 88.3%, SD 9.2%), B (mean 84.7, SD 14.0%), and D (mean 91.4%, SD 7.7%). There was no statistically significant effect of gender (*P*=.84), age (*P*=.22), or education level (lower education level: *P*=.58, middle education level: *P*=.46, versus high education level) on adherence. Mean time of completing the end-of-day symptom diary was 9:46 PM (ie, 14 minutes before receiving the push notification).

### Feasibility of a Smartphone App as Primary Data-Collection Method

Several technical issues were noted by the investigators or reported by patients. In most cases, the cause was found, and the issue was resolved by the app development team without data loss. Encountered hurdles included difficulties installing the app during the screening visit due to connectivity failure, not receiving reminder notifications, inaccurate visual scaling of questionnaires on smaller smartphone screens, updates of Android or iOS operating systems that interfered with prior processes, and connectivity failure due to server maintenance. All documented technical issues and their short-term consequences are presented in Table 2.

**Table 2:** Technical difficulties and consequences with regard to the digital symptom diary.

Description of technical issue	Patients affected, n	Consequence and solution, if applicable
Low internet connectivity hindered installation of the app during the screening visit	15	In most cases, the problem was solved by moving to a location with better internet connectivity or by postponing the installation to a later time.
Not receiving push-notifications as a reminder to complete the end-of-day questionnaires	12	In many cases, patients would complete the questionnaire regardless of receiving the notification. However, the exact effect is unknown, and it may have negatively impacted adherence during days that no notification was received. In most cases, the problem could be resolved by changing the telephone settings (eg by ignoring battery optimizations). In 2 cases in which the issue could not be resolved, reminders were given during the study period by setting the alarm of the device at 10 PM. In the short period during which it was unknown how many devices were affected, additional text messages were sent as reminders.
Incomplete views of the questions due to a too large scaling on smaller smartphone screens.	8	The issue was resolved by adjusting the scaling in the app during updates. Because only a few letters were not depicted correctly and because all participants had received a manual that included the actual questions asked, the negative effect of short-term scaling issues was estimated to be negligible.
iOS or Android updates that interfered with prior settings of the app	0	The issue did not lead to missing data because the small bugs did not shut down the app. The development team provided updates that resolved the issues as soon as possible.
Maintenance of the hosting server	21	The issue led to missing data of one complete day (ie, the day on which the maintenance took place) in all but 2 patients who were included at the time of the maintenance.

### Web-Based Questionnaires: Patients’ Adherence and Completeness

Adherence to the web-based questionnaires was also excellent. One patient did not complete the questionnaires at the end of the treatment period; all others completed all questionnaires until the end of the study or until discontinuation (n=11 discontinued the study). Halfway through the study duration, however, a routing error in one questionnaire became apparent. Although this mistake was corrected immediately, the error had already led to missing data for that particular question in 23.3%-54.0% of all patients, depending on measurement moment (Table S1 in Multimedia Appendix 1). No missing items were found in other questionnaire items.

### Investigators’ Adherence to the eCRF

Adherence of the investigators to the eCRF was excellent with a completion rate of more than 99%. In total, there were 27 patients with at least 1 missing variable in the case report file, 11 of whom discontinued the study during the treatment period (the missing values comprehend follow-up calls that were not conducted). The remaining 17 cases with missing data were because of missed follow-up calls (in 11 cases, 1 follow-up (out of 3) was missed), not registering if additional information about the 6-month follow-up period was given, not registering the date of the last menstruation, not registering if the general practitioner was informed about participation in the study, or not registering the number of capsules that were reported not to be taken during one of the follow-up calls.

## Discussion

The results of this study demonstrate that patients’ adherence to the end-of-day questionnaire in the digital symptom diary was excellent, with a mean completion rate of 87.9% over 70 days of study duration. The total proportion of missing data and data loss due to technical issues of the app was small, indicating that it is safe and realistic to use the app as a primary data-collection method. Furthermore, patients’ adherence to the web-based questionnaires and investigators’ adherence to the eCRF were also outstanding with completion rates of more than 99%.

In terms of electronic diary usage in clinical trials, the adherence rate found in this IBS study is at least comparable to or higher than previously reported rates [5,14,15]. Most people (90.3%) in the Netherlands own a mobile phone [21]. Only a few patients (n=4) needed a device from the investigator team to participate in the study, and only a single patient stated digital data collection as a reason not to participate. Mean adherence to the digital symptom diary decreased by 11% from the first week of the pretreatment period to the last week of the 8-week treatment period (Figure 4). A slight decrease in adherence to the diary during a study period (ie, logging fatigability) is not uncommon and has also been observed in other studies investigating digital diaries [5,22]. Regarding the usage of digital diaries in randomized controlled trials to assess treatment response (according to FDA-recommended definitions) in IBS patients specifically, we are aware of one recent IBS study [23] that applied an electronic diary to assess treatment effect. However, a direct comparison with this study was not possible, as details on the type of device, app, or adherence to the diary were not provided.

With regard to sociodemographic and clinical patient characteristics associated with completing the daily entries in the diary, we found no evidence of a statistically significant effect of gender (*P*=.84), age (*P*=.22), or education level (lower education level: *P*=.58, middle education level: *P*=.46, versus high education level) on adherence. This differs from the results of some prior studies and meta-analysis [15,17] that observed, for example, a statistically significant positive effect of age on adherence. Our interpretation is that this may be caused by the relatively young patient population in the current study. We observed that patients with higher anxiety scores had lower adherence to the digital symptom diary. These data are in line with those of Aaron et al [24], showing that participants with higher stress levels may have lower completion rates. Interestingly, a negative association was found between one inclusion center and adherence. All 4 inclusion centers were located in urban areas but with a wide geographical spread throughout the Netherlands as shown in Multimedia Appendix 1 (Figure S2). The center with the negative association (center C) was in the most urban and populated area (ie, the Amsterdam-The Hague-Rotterdam-Utrecht urban agglomeration). No obvious demographic or baseline differences were observed between study populations in different inclusion centers. No association was found between the lower adherence and the investigator by whom the instructions were given. Although the reason for lower adherence of patients included in this center is unclear, religious and cultural backgrounds of inhabitants of this agglomeration may have differed from those of the inhabitants of other geographical areas [25-27]. Nevertheless, overall adherence during the treatment period in this inclusion center was still good (mean 82.3%, SD 12.5%).

In terms of technical issues arising during the study, minor bugs occurring as a consequence of ever evolving smartphones and operating systems are practically inevitable. It is our experience, therefore, that continuous maintenance and software updating by a development team is crucial to avoid data loss and potential agitation of the study participant due to app malfunctioning. Consequently, the feasibility of using a smartphone app as a primary data-collection method depends to a large extent on skills and availability of development team staff, and research groups should check if appropriate support is available before opting for such methods.

Many high-quality IBS trials have used interactive voice response systems as the primary data collection method [28-31]. In spite of this frequent use, the interactive voice response systems used in IBS trials have not been described in detail, thereby hampering replication and implementation of the methodology in other trials. For comparison with our methodology, we therefore depended on what is known about interactive voice response systems in general. Akin to a digital symptom diary, the interactive voice response systems method allows control of time-windows in which surveys should be completed, provides automated time-stamps to answers, performs data verification and validation, follows a predefined routing schema, enables automatic reminders, collects and stores data in real time, and leads to an overall consistent survey administration. In addition, both methods equally depend on telephone- or internet-service and require staff to program and maintain the software. A potential advantage of interactive voice response systems over those of digital diaries is that it does not depend on literacy skills of the participant. An interactive voice response systems may also need fewer software updates than what is required by smartphone apps due to the high pace of smartphone operating system updates. Potential disadvantages of the interactive voice response systems compared with digital symptom diaries are (1) the inability to get clarification during the survey, whereas a digital symptom diary can have built-in optional clarification of questions; (2) not all interactive voice response systems are equipped with speech recognition; open-ended questions then require transcription by a data manager; (3) the quality of open-ended question recordings depends on enunciation, background noise, and connection; and (4) usage of the interactive voice response systems requires extensive participant training and could be less user friendly [32]. As for patient adherence to the interactive voice response systems, this was reported by only one recent IBS trial [30]; they reported a mean adherence rate of 71% and 73% in the 2 groups examined, when adherence was defined as completing at least 80% of the scheduled calls to the interactive voice response systems. Adherence to the interactive voice response systems in that study [30] was thus notably lower than adherence to the digital symptom diary found in this study.

This study described the overall framework for digitalized data collection used in the PERSUADE study. In addition to the digital symptom diary, the electronic framework used in this drug trial consisted of web-based patient questionnaires and an electronic CRF to collect additional secondary outcomes. A troublesome issue that occurred was a routing error in one of the questionnaires that was discovered too late and had already led to a high proportion of missing data (Table S1 in Multimedia Appendix 1). This applied to only a single question, but routing errors can have potentially disastrous consequences. As such, investigators and data managers should take appropriate care and time when testing questionnaires. Data exports should, furthermore, be examined in an early testing phase and preferably by more than one investigator and data manager. Similar to the diary, the web-based questionnaires and the eCRF featured built-in routing of questions, data validation, and response requirements to encourage data quality and completeness. Overall, these steps allowed for guaranteed standardized data collection with completeness of more than 99% for the web-based questionnaire and eCRF items.

Additional advantages of the combined framework for digitalized data-collection are (1) the ability to monitor patients and their adherence; (2) a reduction in paperwork and physical archiving (eg, in this study the paperwork was reduced to one single informed consent file); (3) manual data transcription can be omitted as research data enter the database immediately; (4) the possibility to adjust and individualize the smartphone app, eCRF, and web-based questionnaires according to the needs of each particular study, and (5) more accurate and standardized data reporting since no error-prone re-entry is necessary. The described framework for digital data collection can, therefore, be employed in different studies investigating different disease entities.

Our findings should be interpreted in light of some potential limitations. First, the study was not primarily designed for the analysis of adherence to the digital symptom diary but for measuring the main clinical outcome [16]. However, since almost 200 patients were included, the sample size was sufficiently large to estimate adherence with enough precision. Second, adherence rate was not assessed within a controlled trial with a more traditional method of data collection (ie, paper-and-pencil diaries, interactive voice response systems) as a comparison. However, the rapid diffusion toward digital approaches in health care and clinical research renders such comparisons less meaningful from a practical point of view as the use of digital techniques become inevitably ubiquitous. In addition, it is unlikely that these traditional approaches to data collection would result in higher adherence than those observed here. Another limitation was that patient satisfaction with the digital diary or web-based questionnaires was not quantified by means of a questionnaire. In this study, the feasibility of the used framework was evaluated primarily on the basis of patients’ and investigators’ adherence and the proportion of complete data, whereas quantified patient satisfaction was not taken into account. However, a low patient satisfaction would have likely led to a lower adherence, and thereby, a higher proportion of missing data. Therefore, although helpful, it is unlikely that applying such a questionnaire would have altered our main findings.

In this IBS drug trial, the use of a smartphone app as a digital symptom diary to assess treatment response was found to be highly feasible and resulted in high quality data collection with excellent patient adherence of more than 86% during the complete study period. The combination of the digital diary with the eCRF, planning tool, and web-based questionnaires led to overall standardized state-of-the art data collection with excellent completeness and can be used as a framework for future randomized controlled trials. Due to the slight decrease in patient adherence to the digital diary throughout the study, caution is needed when using such methods in long-term studies. Although this framework was designed for IBS clinical trials, the results reported here are of added value to a far broader range of disorders for which the collection of patient-reported outcome measures is required. Future studies should preferably include a control group, for example, a group using the interactive voice response system or a group using the app without receiving reminder notifications, to compare adherence and to ascertain specific factors driving high adherence.

## Appendix

Multimedia Appendix 1 Supplementary material.

## Abbreviations

eCRF: electronic case report file
FDA: US Food and Drug Administration
IBS: irritable bowel syndrome
MEMIC: Center for Data and Information Management at Maastricht University
