# Correction: Smart Data Collection for the Assessment of Treatment Effects in Irritable Bowel Syndrome: Observational Study

**DOI:** 10.2196/27998

**Published:** 2021-02-18

**Authors:** Zsa Zsa R M Weerts, Koert G E Heinen, Ad A M Masclee, Amber B A Quanjel, Bjorn Winkens, Lisa Vork, Paula E L M Rinkens, Daisy M A E Jonkers, Daniel Keszthelyi

**Affiliations:** 1 Division Gastroenterology-Hepatology, Department of Internal Medicine NUTRIM School for Nutrition and Translational Research in Metabolism Maastricht University Medical Center+ Maastricht Netherlands; 2 MEMIC Center for Data and Information Management Maastricht University Maastricht Netherlands; 3 Department of Methodology and Statistics Maastricht University Medical Center+ Maastricht Netherlands; 4 Care and Public Health Research Institute Maastricht University Medical Center+ Maastricht Netherlands

In “Smart Data Collection for the Assessment of Treatment Effects in Irritable Bowel Syndrome: Observational Study” (JMIR Mhealth Uhealth 2020;8(11):e19696) the authors noted one error.

The phone number originally published in the Corresponding Author address has been removed from the paper for privacy reasons.

The correction will appear in the online version of the paper on the JMIR Publications website on February 18, 2021, together with the publication of this correction notice. Because this was made after submission to PubMed, PubMed Central, and other full-text repositories, the corrected article has also been resubmitted to those repositories.

