# Supplementary material for: Smart Data Collection for the Assessment of Treatment Effects in Irritable Bowel Syndrome: Observational Study
Source: JMIR Mhealth Uhealth. 2020 Nov 2;8(11):e19696. doi: 10.2196/19696 (PMC7669448; doi:10.2196/19696)
Supplement: Multimedia Appendix 1 [file mhealth_v8i11e19696_app1.docx]

**SUPPLEMENTARY MATERIAL**ORIGINAL PAPER

**Title: Development and evaluation of smart data-collection and its compliance for the assessment of treatment effects in irritable bowel syndrome: an observational study.**

Z.Z.R.M. Weerts, K.G.E. Heinen, A.A.M. Masclee, A.B.A. Quanjel, B. Winkens, L. Vork, P.E.L.M. Rinkens, D.M.A.E. Jonkers, D. Keszthelyi

[METHODS 2](#_Toc38982967)

[1. In- and exclusion criteria 2](#_Toc38982968)

[Eligibility criteria for pre-treatment period 2](#_Toc38982969)

[Exclusion criteria for pre-treatment period 3](#_Toc38982970)

[Inclusion criteria for the actual treatment period 3](#_Toc38982971)

[SUPPLEMENTARY TABLES 5](#_Toc38982972)

[Supplementary Table 1. Proportion of patients with missing data in web-based questionnaires 5](#_Toc38982973)

[SUPPLEMENTARY FIGURES 6](#_Toc38982974)

[Figure S1. Study design of PERSUADE 6](#_Toc38982975)

[Figure S2. Inclusion centers 7](#_Toc38982976)

# METHODS

# 1. In- and exclusion criteria

## **Eligibility criteria for pre-treatment period**

In order to be eligible to participate in the run-in period of this study, subjects must meet all the following criteria:

1. Age between 18 and 75 years;
2. Diagnosed with Irritable Bowel Syndrome according to the Rome IV criteria^26^:
   - Recurrent abdominal pain, at least 1day/week for the last 3 months;
   - Symptom onset at least 6 months prior to diagnosis
     - Associated with two or more of the following:
       1. Pain related to defecation;
       2. Pain associated with a change in frequency of stool;
       3. Pain associated with a change in form (appearance/consistency) of stool;
3. Based on the medical history and previous examination, no other causes for the abdominal complaints can be defined. Especially no history of:
4. Inflammatory Bowel Disease;
5. Celiac Disease;
6. Thyroid dysfunction (if not well-regulated);

If alarm symptoms (including unexplained rectal blood loss or weight loss) are present, a colonoscopy has been performed and was negative for other causes.

1. Women in fertile age (<55 years old) must use contraception or be postmenopausal for
   at least two years.

## **Exclusion criteria for pre-treatment period**

A potential subject who meets any of the following criteria will be excluded from participation in this study:

1. Insufficient fluency of the Dutch language;
2. Any previous use (also incidental use) of peppermint oil capsules in the last 3 months prior to inclusion (the use of peppermint tea, menthol candy etc. is allowed);
3. The inability to stop regular use of medication affecting the gastro-intestinal system (such as Non Steroidal Anti Inflammatory Drugs (NSAID), laxatives, prokinetics, opioids, smasmolytics and anti-diarrhoeal drugs). This use should be halted at least 1 week before enrollment into the run-in period;
   1. The use of 1 antidepressant drug is allowed, providing dosing has been stable for > 6 weeks before enrollment;
   2. The use of 1 proton pump inhibitor (PPI) is allowed, providing dosing has been stable > 6 weeks before enrollment;
4. Previous major abdominal surgery or radiotherapy interfering with gastrointestinal function:
   1. Uncomplicated appendectomy, cholecystectomy and hysterectomy allowed unless within the past 6 months;
   2. Other surgery upon judgment of the principle investigator;
5. History of liver disease, cholangitis, achlorhydria, gallstones or other diseases of the gallbladder/biliary system;
6. Pregnancy, lactation;
7. Using drugs of abuse;
8. Known allergic reaction to peppermint.

## **Inclusion criteria for the actual treatment period**

In order to be eligible to participate in this study, subjects must meet all of the following criteria:

1. No changes in in- and exclusion criteria for the run-in period have occurred;
2. Average worst abdominal pain score (on 11-point NRS) of > 3, during the 14-days pre-treatment period.

# SUPPLEMENTARY TABLES

| **Supplementary Table 1. Proportion of patients with missing data in web-based questionnaires** | |
| --- | --- |
|  | ***N* = 189** |
| *Missingness of all questionnaires due to discontinuation or non-response, N (%)*  Baseline  T = 4 weeks  T = 8 weeks  T = 3 months  T = 6 months | 0 (%)  6 (3.2)  12 (6.3)  11 (5.8)  11 (5.8) |
| *Missingness in question regarding presenteeism due to a routing error, N (%)*  Baseline  T = 4 weeks  T = 8 weeks  T = 3 months  T = 6 months | 102 (54.0)  95 (50.3)  86 (45.5)  69 (36.5)  44 (23.3) |

# SUPPLEMENTARY FIGURES

## **Figure S1. Study design of PERSUADE**

##
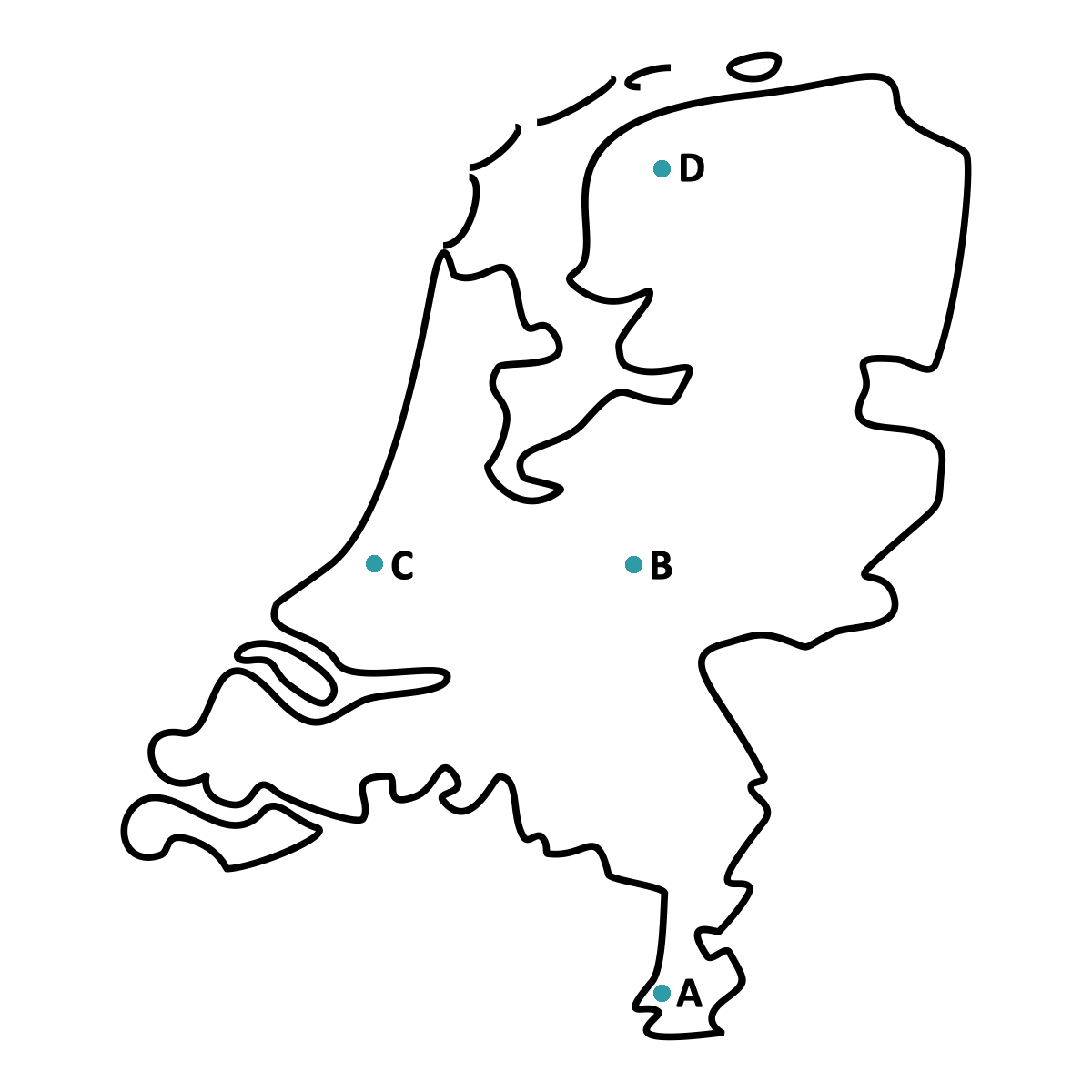
**Figure S2**. **Inclusion centers**

***Supplementary Figure 2.*** The four inclusion centers (hospitals) of the PERSUADE study were located in the Netherlands in: A, Maastricht; B, Ede; C, Leiden; D, Leeuwarden.
